# Genetic Features of Late Onset Primary Hemophagocytic Lymphohistiocytosis in Adolescence or Adulthood

**DOI:** 10.1371/journal.pone.0107386

**Published:** 2014-09-18

**Authors:** Yini Wang, Zhao Wang, Jia Zhang, Qing Wei, Ran Tang, Junyuan Qi, Lihong Li, Liping Ye, Jijun Wang, Ling Ye

**Affiliations:** 1 Department of Hematology, Beijing Friendship Hospital, Capital Medical University, Beijing, China; 2 Department of Lymphoma, Institute of Hematology and Blood Diseases Hospital Chinese Academy of Medical Sciences, Tianjin, China; 3 Department of Hematology, Beijing Chao-Yang Hospital, Capital Medical University, Beijing, China; 4 Department of Transplantation, The 309th Hospital of Chinese People's Liberation Army, Beijing, China; 5 Department of Hematology, Peking University Third Hospital, Beijing, China; 6 Department of Hematology, Shandong Jining First People's Hospital, Shandong, China; University of Cape Town, South Africa

## Abstract

Hemophagocytic lymphohistiocytosis (HLH) is a life-threatening condition of uncontrolled immune activation leading to extreme inflammation. Primary HLH was once believed to be a disease that occurred only in infancy or young children, and was rarely diagnosed in adults. It is now understood that patients can develop primary HLH in their adolescence or adulthood. This study included 252 adolescent and adult patients with a clinical diagnosis of HLH from 35 general medical institutions across mainland China. All exons and 50 bp of flanking intronic sequence of six HLH-related genes (PRF1, UNC13D, STX11, STXBP2, SH2D1A, and BIRC4) were sequenced in these patients. We identified mutations in 18/252 (7.1%) of the patients, with changes in PRF1 being most common. Late-onset HLH often features viral infection and other predisposing factors. We conclude that late-onset primary HLH is not as rare as previously thought. Older patients should not be delayed to receive HLH-related genes testing when they are suspected with HLH.

## Introduction

Hemophagocytic lymphohistiocytosis (HLH) is an immune disorder characterized by the uncontrolled activation of T lymphocytes and macrophages and the excessive generation of inflammatory cytokines. The disease presents clinically with persistent fever, hepatosplenomegaly, pancytopenia, and hemophagocytic phenomena detected in the bone marrow, liver, spleen, and lymph tissue, with onset typically occurring in the first 2 years of life. Primary HLH (also called familial HLH) refers to cases in which there is a positive family history or a defined genetic cause. An onset of primary HLH after 8 years of age has rarely been reported [1]. A hallmark of the disease is decreased NK cell and cytotoxic T lymphocyte (CTL) function, which is thought to be caused by genetic defects in the perforin/granzyme-mediated cytotoxic pathway. Uncontrolled immune activation leading to extreme inflammation causes the onset of HLH symptoms [2]. Untreated HLH disease progresses rapidly and has a high mortality rate. The HLH-94 treatment protocol involves the use of corticosteroids, cyclosporine A, and etoposide before hematopoetic stem cell transplant (HCT) [3].

Since 1975 when Chandra [4] reported adult patients with clinical features similar to primary HLH brought on by infection, HLH in older children and even adults has generally been called secondary HLH. It is now understood that patients with a genetic basis for HLH can sometimes remain asymptomatic until adolescence or even adulthood [5]–[7]. Recently, Zhang et al. reported twenty-five adult patients with HLH possessed genetic mutations in North America and Sieni et al. described eleven adult FLH in Italy [8], [9]. Various mutations in at least 9 genes have been shown to play roles in primary HLH [10]–[19]. PRF1, UNC13D, STX11, STXBP2, RAB27A, LYST, and AP3B1 are autosomal genes, all involving perforin/granzyme-mediated cytotoxicity. SH2D1A and BIRC4 are X-linked genes, coding for an adaptor molecule called signaling lymphocyte activation molecule-associated protein (SAP) which is involved in signaling pathways that trigger cytotoxic granule release, and more recently, X-linked inhibitor of apoptosis. More importantly, all cases of HLH, regardless of genetic involvement, require prompt diagnosis and initiation of treatment.

The aims of this study were to characterize genetic changes in a large population of adolescents and adults with HLH. The Beijing Friendship Hospital, Capital Medical University accepts specimens and referral cases from all over the country and has the largest adult HLH patient database in mainland China. For this study, we sequenced all coding exons and at least 50 base pairs of the adjacent intronic sequence of the PRF1, UNC13D, STX11, STXBP2, SH2D1A and BIRC4 genes in 252 adolescent and adult HLH patients from mainland China. We also measured NK cell activity to see if it correlated with certain mutations.

## Methods

A total of 252 adolescents and adults (age ≥13 years) diagnosed clinically with HLH were ascertained between July 2006 and June 2012. The patients were diagnosed according to HLH-2004 diagnostic criteria [20] and referred to our institution from 35 general medical institutions across China (see Acknowledgments). Clinical data and peripheral blood samples were provided for each patient by their referring physician. NK cell function was determined by the lactate dehydrogenase (LDH) release assay [21], [22]. Normal values for adolescents and adults in our lab range from 31.54% to 41.58% [21].

Total genomic DNA was isolated from peripheral blood samples of each patient with the MagaZorb DNA Kit (Promega, Madison, WI, USA), according to the manufacturer's instructions. All coding exons and at least 50 base pairs of the adjacent intronic sequence of the six genes associated with HLH (PRF1, UNC13D, STX11, STXBP2, SH2D1A, and BIRC4) were PCR amplified and sequenced. The patient sequences were compared to those published in Genbank (PRF1, NM_005041.4; STX11, NM_003764.3; SH2D1A, NM_002351.3; UNC13D, NM_199242.2; BIRC4, NM_001167.2; STXBP2, NM_006949.2).

The ethics committee of Beijing Friendship Hospital approved this study. Written informed consent was given by the patients and their caretakers or guardians for their clinical and genetic information to be stored in the hospital database and used for research.

## Results

This study group included 252 adolescent and adult patients diagnosed with HLH (147 males, age range 13–81 years). Patients were grouped by age of onset. There were 57 adolescent patients aged 13 to 17 years, and 195 adult patients aged 18 years and older. The entire coding regions and flanking intronic sequence of six genes (PRF1, UNC13D, STX11, STXBP2, SH2D1A, and BIRC4) were PCR amplified and sequenced for each patient. We identified a gene sequence change in 18 of the 252 patients (7.1%). These 18 patients (13 males and 5 females, 13–56 years) included 8 adolescents and 10 adults, accounting for 14.0% and 5.1%, respectively, of patients the same age group. Twelve of these patients had viral infections, including EBV, CMV, and HHV-7. No patients in this study had a family history of HLH disease.

Table 1 lists the clinical and DNA findings for the 18 patients with identified genetic changes. PRF1 mutations were the most common. We found 12 different PRF1 gene changes among nine patients in whom a gene change was identified, including a nonsense mutation (p.Q166X), a frameshift mutation (p.P22RfsX29), and 10 missense mutations, all at different sites. Five of the nine PRF1 mutation carriers were compound heterozygous for the PRF1 mutations. Two missense mutations in the STX11 gene (p.R49Q and p.R49W) were identified in seven patients. A single missense mutation in SH2D1A (p.A3S) was identified in two patients, and one patient had a UNC13D missense mutation. No changes in STXBP2 or BIRC4 were found in any of the 252 HLH patients in this study.

**Table 1:** Clinical and genetic findings for 18 HLH patients with identified mutations in HLH-related genes.

Group	Patient	Age	Sex	Gene sequencing results	NK cell activity (%)	sCD25 (IU/mL)	Treatment response	Outcome	Last follow-up time(Month)
**Biallelic or hemizygous mutations**	P01	13	F	PRF1: c.503G>A(p.S168N) c.1177T>C(p.C393R)	19.05	5812	Remission/HSCT	Survival	36
	P03	13	M	PRF1: c.673C>T(p.R225W) c.1304C>T(p.T435M)	10.58	4547	No remission/HSCT	Death	8
	P04	27	M	PRF1: c.127C>A(p.L43M) SH2D1A: c.7G>T(p.A3S)	9.82	3270	Remission/HSCT	Survival	12
	P06	15	M	PRF1: c.65delC(p.P22RfsX29) c.1349C>T(p.T450M)	9.14	3822	Remission/HSCT	Survival	18
	P09	46	M	PRF1: c.10C>T(p.R4C) c.98G>A(p.R33H)	6.95	11419	Remission	Survival	8
	P12	18	F	SH2D1A: c.7G>T(p.A3S)	18.46	3276	No remission	Death	12
	P14	54	F	PRF1: c.65delC(p.P22RfsX29) c.674G>A(p.R225Q)	7.14	10562	Remission	Survival	8
**Monoallelic mutations**	P02	48	M	PRF1: c.916G>A(p.G306S)	20.13	12277	No treatment	Death	5
	P05	56	M	STX11: c.146G>A(p.R49Q)	27.15	7422	No remission	Death	8
	P07	53	M	PRF1: c.65delC(p.P22RfsX29)	14.26	8615	Remission	Relapse/Remission	42
	P08	14	F	STX11: c.146G>A(p.R49Q)	23.50	4131	No remission	Death	6
	P10	22	M	PRF1: c.496C>T(p.Q166X)	26.75	4824	Remission	Relapse/Death	14
	P11	42	M	STX11: c.146G>A(p.R49Q)	16.12	9328	No remission	Death	7
	P13	14	M	STX11: c.146G>A(p.R49Q)	10.73	4604	Remission	Survival	12
	P15	16	M	UNC13D: c.1120C>A(p.P374T)	9.82	8964	Remission/HSCT	Survival	23
	P16	16	F	STX11: c.145C>T(p.R49W)	1.76	5631	Remission	Survival	18
	P17	15	M	STX11: c.146G>A(p.R49Q)	26.91	733	Remission	Survival	21
	P18	36	M	STX11: c.146G>A(p.R49Q)	17.77	8019	Remission	Survival	32

Normal NK cell activity range: 31.54–41.58%.

Normal sCD25 value: <2400 IU/mL.

Both patients with the SH2D1A p.A3S mutation are noteworthy. One male patient (P04) also had a PRF1 mutation. Because SH2D1A p.A3S is X-linked, this mutation was inherited from his mother, and the PRF1 mutation was inherited from his father. The SH2D1A mutation is X-linked recessive, and the PRF1 mutation is autosomal recessive, thus the SH2D1A change is thought to play a dominant role in the pathogenesis in this particular patient. The other patient with the SH2D1A p.A3S mutation (P12) was a female. Analysis of her parents showed that she inherited her abnormal X from her asymptomatic father.

Of the 18 patients in whom a mutation was identified, 11 had monoallelic mutation identified. In all patients, the NK cell activity was lower than normal levels. The NK cell activity was slightly lower in patients with biallelic mutation (11.59±5.07%) than in patients with monoallelic mutation (17.72±8.21%), although the difference was not statistically significant (*P* = 0.097). Soluble CD25 (sCD25) levels, a marker of inflammation and disease status, were similar between patients with biallelic mutation (6101±3461 IU/ml) and those with monoallelic mutation (6777±3166 IU/ml), (*P* = 0.68).

**Figure 1:**
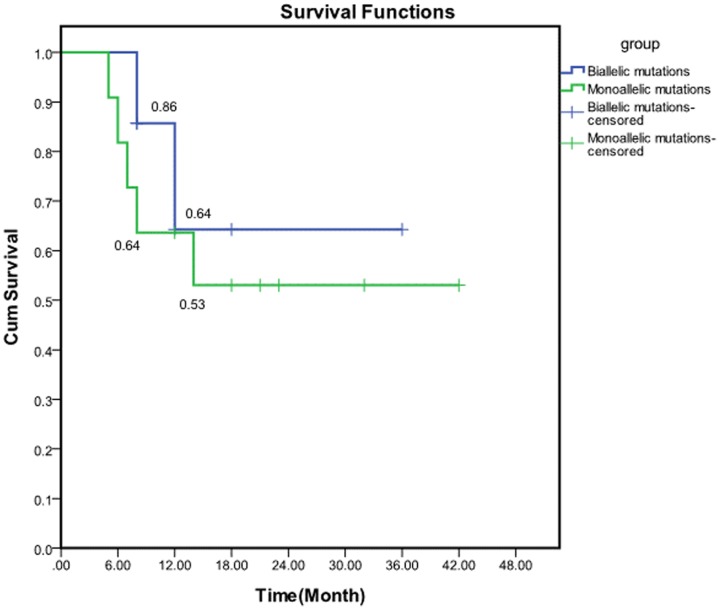
Comparison of the survival rate in the two groups.

Seventeen of the 18 patients presented in this study received HLH-94 drug treatment. The patient who refused treatment died soon after diagnosis. Twelve patients achieved clinical remission after the first treatment, while five patients died without remission. Two of 12 remission patient relapsed, one died and the other achieved re-remission. Four of them underwent allogeneic HCT after first remission and survived disease free. At the termination date of the follow up, seven patients died, and eleven patients survived, with a total survival rate of 61%. In the biallelic mutation group, two patients died and five survived. In the monoallelic mutation group, five patients died and six survived. There was no significant difference in the survival rate between the two groups (*P* = 0.53). Figure 1 shows the survival function of the two groups.

## Discussion

Primary HLH was first proposed by Farquhar in 1952, who described high fever, pancytopenia, hepatosplenomegaly, and rapid death in 2-month-old twins [23]. In subsequent reports, almost all patients were infants and young children, and more than half of the patients were twins, suggesting a genetic basis for the disease. Until a perforin gene mutation was found in a case of familial HLH [10], the diagnosis of primary HLH required onset in infancy and a positive family history as the support basis. After several additional genes and mutations were found to be associated with HLH, the Histiocyte Society developed the diagnostic guidelines HLH-2004. Evidence of a genetic defect is one option for diagnosing primary HLH; however, patients with refractory/reactivated HLH should also be considered as patients with severe disease. Adult HLH patients are typically diagnosed with secondary HLH because the disease seems to be induced by a concomitant condition, such as EBV infection, malignancy, or rheumatologic disorders [24]. However, many of the genetic changes found in the pediatric primary HLH cases are also reported in adults [8]. Here, we report genetic findings from a study of 252 adolescent and adult onset HLH patients from mainland China.

Mutations in PRF1 occur in 15% to 50% of patients with primary HLH [25]. Similarly, we found a PRF1 mutation in 9 of 18 (50%) patients in whom a mutation was identified. PRF1 is located at 10q21–22 and has 3 exons, with all coding sequences in exons 2 and 3. It has been clearly documented that PRF1 mutations cause decreased or absent perforin protein expression on the surface of cytotoxic cells. This condition prohibits cytotoxic and NK cells from destroying their target cells, which in turn leads to increased cytokine production and macrophage activation, causing the symptoms of HLH [26].

Cases of primary HLH with PRF1 mutations have been reported worldwide. At least 70 different mutations have been described between the two coding exons of PRF1, with p.W374X being the most common. Geographical and racial distributions of various PRF1 mutations have been reported [27]. It is possible that the different PRF1 mutations influence clinical severity. The mutation p.A91V, for example, occurs in a functionally unimportant region of the protein and, therefore, may lead to mild symptoms or delayed onset of HLH. Mutations causing slight reductions in perforin expression and NK cell function could be the basis of HLH that is dormant until triggered by external factors such as infection [6].

We identified two patients in our study with X-linked SH2D1A gene sequence abnormalities, one of them was a heterozygous female carrier (P12, Table 1) who experienced onset of HLH clinical symptoms at age 18, associated with T-cell lymphoma. Analysis of her parents revealed that the abnormal X chromosome came from her asymptomatic father. Generally, this mutation is predicted by in-silico analysis (SIFT, Poliphen) to be tolerated. We think that the onset of HLH disease in the carrier female may be due to nonrandom X-inactivation or differences in methylation that allow expression of the abnormal SH2D1A gene. Viral infection, particularly EBV infection, is an important predisposing factor in HLH. Studies have shown that individuals with immune deficiencies such as X-linked lymphoproliferative syndrome (XLP) are usually asymptomatic before EBV infection, and there is no clear genotype/phenotype correlation [28]. The onset and severity of HLH disease may differ among individuals due to varying exposure to triggering factors. In the absence of such triggers, the disease gene carrier may have no clinical manifestations, which may explain the phenomenon we observed in the female X-linked SH2D1A mutation carrier and her father. In the other hand, the HLH in these two patients may also be secondary to EBV infection or lymphoma.

In this study, we observed 12 different PRF1 mutations in nine patients, most of whom had viral infection at HLH onset. Their diagnosis of secondary HLH was revised to primary HLH after genetic testing. The 7 patients with STX11 mutations also had viral infection that seemed to trigger HLH disease onset. Suppression of life-threatening inflammation must be initiated promptly in cases of both primary and secondary HLH. However, the long-term treatment strategies should focus on the underlying cause of HLH for patients in these two categories. For secondary HLH patients, control of the triggering disease or infection is critical for preventing recurrence. For primary HLH patients, who have a genetic mutation causing an immune deficiency, restoration of the lacking immune system components is vital. Therefore we believe that a clear diagnosis of primary or secondary HLH is important for long term therapy. HLH-related genetic testing can aid in distinguishing HLH types and subsequent treatment. And the functional studies, such as degranulation assays, are of great importance and should be implemented by all laboratories serving as prospective referral [29].

In this study of 252 adolescent and adult HLH patients, the overwhelming majority had no identifiable mutation in the six HLH-related genes sequenced. We identified monoallelic mutation in 11 patients, and biallelic mutation in seven additional patients. Therefore, more than half of patients with identifiable genetic changes carried only one mutation in one of these genes. It is possible that other types of mutations were not detectable by the methodology used in this study, or that these patients possessed mutations in additional genes that have not yet been discovered to be associated with HLH. NK cell activity was lower than normal in each patient, and there was no statistically significant difference between monoallelic mutation group and biallelic mutation group. Our results support that defective cytotoxicity might be involved also in a proportion of HLH without biallelic mutations in the FHL related genes. Our data and other similar findings strongly suggest that “milder” mutations in these genes play an important role in the development of late-onset HLH when patients are challenged by viral infection or other environmental stresses.

Nowadays, HLH remains a rapidly progressive and life-threatening disease. Adoption of the HLH-94 treatment protocol increased the 3-year survival rate of HLH patients from less than 10% to about 50% [3]. In our study, the initial remission rate was 67%, and the total survival rate was 61%. The clinical results presented here are similar to others. But there was no significant difference in the survival rate between monoallelic mutation group and biallelic mutation group in our study (*P* = 0.536). However, this conclusion needs to be checked up with large scale clinical study.

With the recognition of HLH in adolescent and adult patients, late-onset primary HLH occurs more commonly than was suspected previously. Furthermore, patients with recurrence of HLH, in the absence of autoimmune disease or malignancy, are most likely to be suffering from primary HLH. Older patients should receive HLH-related genetic testing when HLH is suspected, since a proper diagnosis can aid in long term care and prevention of recurrence.
